# Effectiveness of sertraline in treatment of depression in a consecutive sample of patients with acute myocardial infarction: six month prospective study on outcome

**DOI:** 10.1186/1745-0179-1-26

**Published:** 2005-12-09

**Authors:** Prasanta Kumar Mohapatra, Nilamadhab Kar, Gopal Chandra Kar, Mrutyunjaya Behera

**Affiliations:** 1 Consultant Psychiatrist, Mental Health Institute, SCB Medical College, Cuttack, India; 2 Consultant psychiatrist, Corner House Resource Centre, 300, Dunstall Road, Wolverhampton, WV6 0NZ, UK; 3 Professor and Head of Department of Psychiatry, Mental Health Institute, SCB Medical College, Cuttack, India; 4 Professor and Head of Cardiology, SCB Medical College, Cuttack, India

## Abstract

**Background:**

A considerable proportion of patients suffer from depression following acute myocardial infarction. Information regarding this prevalence in Indian patients and response to treatment is lacking.

**Method:**

Prevalence of major depression was studied in 50 consecutive in-patients with acute myocardial infarction following DSM-IV criteria. In a comparative study outcome of treatment with sertraline in terms of recovery from depression, change in Hamilton Depression Rating scale score and occurrence of cardiac events were ascertained for a 6-month period, which were compared with a group of patients who did not receive sertraline.

**Results:**

Major depression was diagnosed in 34% of patients. Female patients were more affected than the males. Hamilton Depression Rating scale scores gradually decreased in both the groups over the study period; however it became significantly less for patients treated with sertraline. Significantly more number of patients who received sertraline recovered from depression. There were no dropouts due to non-response or side effects. Cardiac events were reported less in this group than those who continued only cardiac treatment as usual.

**Conclusion:**

A considerable proportion of acute myocardial infarction patients have major depression. Sertraline is efficacious and safe in these patients. Antidepressant treatment might be associated with reduction of cardiac events.

## Introduction

Sadness is often portrayed as a feeling of heaviness in the chest or as 'broken heart' [1]; and the relation has been a subject of both literature and science. Symptoms of depression in patients with acute myocardial infarction (AMI) have been reported from 8 – 45% [2], up to 65 percent [3]. Prevalence of major depression range from 10 to 37.7% in various reports [3-8]. Prevalence rates of 30.9%, 37.7% and 37.2% during hospitalization, and 4 and 12 months later respectively have been reported [7]. There is no available data on this from Indian population. Prevalence from neighboring Asian country Pakistan suggest symptoms of depression in 14%, and mixed anxiety and depression symptoms in 18% of the patients following AMI [9]. The prevalence figures of depression following AMI are considerably higher than the general population.

The presence of depression confers significant risks for additional cardiac morbidity and mortality in patients with coronary heart disease [4]. It increases hospital length of stay, procedures, readmission rates, and the cost of medical care [10]. It is associated with an increased risk of re-infarction [4]; and is known to be an independent risk factor for death after AMI [11,12]. In addition, major depression at the time of diagnostic coronary angiographies more than doubles the risk of a major cardiac event within one year [13]. Depression, even after adjustment for potential confounders has been associated with significant worsening of quality of life in cardiac patients [14]. It predicts slow recovery and a poor quality of life following AMI [15].

In spite of the fact that depression is an independent risk factor additional care is rarely taken of depressed acute myocardial infarction patients largely due to a failure in identification [11]. With above background, considering the facts that there is paucity of Indian data on this subject, we studied the prevalence of depression in AMI in-patients. We also tried to find out the effect of antidepressant treatment on the cardiac events up to 6 months, in a prospective study.

## Methods

The study was conducted in the departments of Cardiology and Psychiatry of S.C.B. Medical College, Cuttack. Consecutive patients suffering from acute myocardial infarction, admitted to the cardiology ward were included in the study. Patients, who were recovering from by-pass surgery; patients having history of depressive disorders before the onset of cardiac problem, current substance harmful use or dependence which could contribute to mood status; and those who are already receiving psychotropic medications, were excluded.

The patients were randomized as treatment as usual group or intervention group before being evaluated. The process followed a design where participants are randomized to control or intervention before consent is sought [16]. The randomization was done by the cardiologist and one psychiatrist (GK), and the rater (PM) remained blind to the status of the participants. Informed consent was obtained. All patients were clinically evaluated and diagnosed according to Diagnostic and Statistical Manual of Mental Disorders-IV [17]. Hamilton rating scale for depression [18] (HDRS) were administered to asses the degree of depression in the depressed patients.

Socio-demographic information and risk factors (hypertension, diabetes mellitus, dislipidaemia, history of smoking and alcohol abuse, and family history of coronary artery disease (CAD) were recorded.

All the patients who were diagnosed to have depression and were in the intervention group were offered antidepressant treatment. They were prescribed only sertraline, considering reports of its greater tolerability in cardiac patients [19]. The dose of sertraline was titrated to a range of 50 to 200 mg per day depending upon clinical evaluation. Patients in 'treatment as usual' (TAU) group continued to receive cardiological treatment as required, but they had no antidepressant or other psychotropic medication. Patients in this group, who received antidepressant medication during the study from their cardiologists or other sources, were excluded from the study with the last observation carried forward. Neither group received psychotherapeutic intervention for depression.

HDRS was repeated every month for period of six months. The remission was defined as a HDRS score of 7 or less [20]. One of the psychiatrists (GK) monitored the antidepressant treatment and side effects. Cardiac status was monitored by the cardiologist and the cardiological events viz. recurrent angina, re-infarction, re-hospitalization, heart failure, and death due to cardiac cause were recorded. Analysis was intention to treat. The study was approved by the ethical committee of Mental Health Institute, SCB Medical College, Cuttack.

## Results

Fifty consecutive patients (38 males and 12 females) were enrolled in the study. Depression was found in 17 (34%) patients. Prevalence of depression in various clinical groups is given in table one. Mean age of the whole sample was 58.1 ± 10.2; for those with depression it was 55.8 ± 11.4; which was not significantly different from that (59.2 ± 9.7) of non-depressed patients.

**Table 1:** Prevalence of depression in different socio-demographic and clinical groups

Sociodemographic and clinical variables		Total (n = 50)	With depression (n = 17)	%
Gender	Male	38	10	26.3
	Female*	12	7	58.3
Habitat	Rural	25	9	36.0
	Urban	25	8	32.0
Economic Status	Low	18	6	33.3
	Middle	31	11	34.3
	Upper	1	0	0.0
Clinical risks	Hypertension	19	8	42.1
	Diabetes mellitus	18	6	33.3
	Family History of CAD	10	5	50.0
	History of smoking	15	6	40.0
	History of alcohol abuse	4	2	50.0

% within the variable, * chi square: 4.16; 1, p = 0.041

Prevalence of depression in many socio-demographic variables and risk groups were comparable except that significantly more females were depressed than males. All were married and living with their spouses. None had dislipidaemia.

Table 2 shows socio-demographic profile and clinical characteristics in intervention group and treatment as usual groups. The groups were comparable including mean age (tau group: 56.8 ± 10.8 and intervention group: 55.2 ± 12.1) and the HDRS scores (tau 16.67 ± 7.06 and intervention group 15.0 ± 5.33). In the associated risk factors both the groups were also comparable except that 45% patients in the intervention group receiving antidepressants had family history of CAD, compared to none in the tau group.

**Table 2:** Comparison of Patient Characteristics in Intervention and TAU Group

Sociodemographic and clinical variables		TAU (n = 6)		Intervention (n = 11)	
		n	%	n	%
Gender	Male	4	66.7	6	45.5
	Female	2	33.3	5	54.5
Habitat	Rural	5	83.3	4	36.4
	Urban	1	16.7	7	63.6
Economic Status	Low	3	50.0	3	27.3
	Middle	3	50.0	8	72.7
Clinical risks	Hypertension	4	66.7	4	36.4
	DM	1	16.7	5	45.5
	Family History of CAD*	0	0.0	5	45.5
	History of smoking	3	50.0	3	27.3
	History of alcohol abuse	1	16.7	1	9.1

% within the treatment group; * Chi square: 3.86, df = 1, p = 0.049

It was observed that HDRS scores gradually decreased in both the groups over the study period, however it remained significantly (t: 3.09, df = 15, p = 0.007) more for the tau group (12.0 ± 5.76) in comparison with intervention group (4.82 ± 3.84). At the end of six months ten (90.9%) patients in intervention group achieved remission compared to two (33.3%) of the tau group. The number needed to treat was around 2 (1.74). There were no dropouts due to non-response or side effects. Cardiac events were around 2.8 times more in the tau group compared to the intervention group.

## Discussion

The focused objective of the study was to find out the prevalence of depression in patients with acute myocardial infarction in an Indian population where such data are not available; and to compare the response to antidepressant treatment between those who received it and those who continued treatment as usual without it.

There are a few limitations of the study which need to be highlighted while discussing the results. The sample size was small. Universe of the study was in-patients at the time of inclusion, so the prevalence may not be generalisable to outpatients. Severity of cardiac status was not obtained in a structured way. Patients undergoing angiography have been reported to be a specific risk group however that information was not noted. Other factors contributing or related to depression for example psychosocial issues and family history of depression were not studied.

The prevalence of depression in patients with acute myocardial infarction as observed in the index study (34%) is comparable to that reported elsewhere [3-8]. One of the many reasons for the variability in the prevalence could be the different methodologies and diagnostic approach. It may be highlighted that sample of the index study were in-patients recovering from acute myocardial infarction who did not have past history of depression, or current substance use that can influence mood. This suggests that the prevalence rate is more closely related to AMI. There was no difference in age, rural or urban habitat, socioeconomic status, associated risk factors amongst the depressed and non-depressed. Significantly more females were depressed than males; which reaffirms their greater vulnerability for depression.

Antidepressant treatment group and tau group were comparable considering age, gender and other socio-demographic variables studied. Sertraline has been found to be cost effective strategy in this group of patients [21] making it more relevant to choose. At the end of six months the outcome regarding the remission of depression was significantly different between the two groups. Group HDRS mean, which was comparable at the baseline, was significantly less in the intervention group. In addition there were no dropouts. Sertraline treatment was efficacious and well tolerated similar to reported results [19].

It is known that presence of depression confers significant risks for additional cardiac morbidity and mortality in patients with CAD [1,4,22]. It is associated with an increased risk of re-infarction and mortality following acute infarction [4]. In the index study frequency of cardiological events of both groups was considerably different during the six-month study period; the group receiving treatment had much less cardiological events. Treatment of depression and reduction in cardiac morbidity might be connected, and if this is convincingly proven, it would establish a therapeutic advance in this group of patients [23].

## Conclusion

Prevalence of depression in myocardial infarction patients being treated in the hospital was found to be 34%. Female patients were more affected than the males. Sertraline was safe and efficacious in AMI patients with depression. Less number of cardiac events was reported in depressed patients who received antidepressant treatment during the six-month study period.

## Competing interests

The author(s) declare that they have no competing interests.

## Authors' contributions

PM conceptualized, did literature survey, wrote protocol, collected data, and wrote first draft; NK conceptualized, reanalyzed data, did further literature survey, critically evaluated and revised the article, GK helped in evaluation of patients, monitored treatment, MB helped in collection of cardiological data. All authors read and approved the final draft.

**Table 3:** The outcome at the end of six months

Outcome of depression and cardiac events	TAU (n = 6)		Intervention (n = 11)	
	n	%	n	%
Remission from depression*	2	33.3	10	90.9
				
Cardiac events within study period				
Recurrent angina	4	66.7	5	45.5
Myocardial infarction	4	66.7	2	18.2
Re-hospitalizationfor cardiac reasons	5	83.3	4	36.4
Congestive Heart Failure	3	50.0	0	0.0
Death	1	16.7	0	0.0

* Chi square: 6.19, df = 1, p = 0.013
